# Flow-Through Amperometric Biosensor System Based on Functionalized Aryl Derivative of Phenothiazine and PAMAM-Calix-Dendrimers for the Determination of Uric Acid

**DOI:** 10.3390/bios14030120

**Published:** 2024-02-23

**Authors:** Dmitry Stoikov, Alexey Ivanov, Insiya Shafigullina, Milena Gavrikova, Pavel Padnya, Igor Shiabiev, Ivan Stoikov, Gennady Evtugyn

**Affiliations:** 1 Alexander Butlerov Institute of Chemistry, Kazan Federal University, 18 Kremlevskaya Street, Kazan 420008, Russia; dmistojkov@kpfu.ru (D.S.); inzshafigullina@kpfu.ru (I.S.); magavrikova@stud.kpfu.ru (M.G.); padnya.ksu@gmail.com (P.P.); shiabiev.ig@yandex.ru (I.S.); ivan.stoikov@mail.ru (I.S.); gennady.evtugyn@kpfu.ru (G.E.); 2 Analytical Chemistry Department, Chemical Technology Institute, Ural Federal University, 19 Mira Street, Ekaterinburg 620002, Russia

**Keywords:** flow-through analysis, chronoamperometry, electrochemical biosensor, replaceable reactor, uricase, PAMAM-calix-dendrimers

## Abstract

A flow-through biosensor system for the determination of uric acid was developed on the platform of flow-through electrochemical cell manufactured by 3D printing from poly(lactic acid) and equipped with a modified screen-printed graphite electrode (SPE). Uricase was immobilized to the inner surface of a replaceable reactor chamber. Its working volume was reduced to 10 μL against a previously reported similar cell. SPE was modified independently of the enzyme reactor with carbon black, pillar[5]arene, poly(amidoamine) dendrimers based on the *p-tert*-butylthiacalix[4]arene (PAMAM-calix-dendrimers) platform and electropolymerized 3,7-bis(4-aminophenylamino) phenothiazin-5-ium chloride. Introduction of the PAMAM-calix-dendrimers into the electrode coating led to a fivefold increase in the redox currents of the electroactive polymer. It was found that higher generations of the PAMAM-calix-dendrimers led to a greater increase in the currents measured. Coatings consisted of products of the electropolymerization of the phenothiazine with implemented pillar[5]arene and PAMAM-calix-dendrimers showing high efficiency in the electrochemical reduction of hydrogen peroxide that was formed in the enzymatic oxidation of uric acid. The presence of PAMAM-calix-dendrimer G2 in the coating increased the redox signal related to the uric acid assay by more than 1.5 times. The biosensor system was successfully applied for the enzymatic determination of uric acid in chronoamperometric mode. The following optimal parameters for the chronoamperometric determination of uric acid in flow-through conditions were established: pH 8.0, flow rate 0.2 mL·min^−1^, 5 U of uricase per reactor. Under these conditions, the biosensor system made it possible to determine from 10 nM to 20 μM of uric acid with the limit of detection (LOD) of 4 nM. Glucose (up to 1 mM), dopamine (up to 0.5 mM), and ascorbic acid (up to 50 μM) did not affect the signal of the biosensor toward uric acid. The biosensor was tested on spiked artificial urine samples, and showed 101% recovery for tenfold diluted samples. The ease of assembly of the flow cell and the low cost of the replacement parts make for a promising future application of the biosensor system in routine clinical analyses.

## 1. Introduction

In recent years, electrochemical enzyme biosensors have been widely used for the analysis of food industry facilities [1] in the monitoring of environmental pollution, e.g., pesticides [2], heavy metal ions [3], and other toxins in fresh and waste waters. Enzymatic methods of analysis are also broadly applied in biochemistry and medicine to determine drugs [4] and clinical markers [5,6] in blood and other biological fluids. The peculiarity of enzymes as elements of biological recognition stems from their high selectivity, and the selectivity of enzymes as biorecognition elements, in comparison with other catalysts, make them attractive in the determination of bioactive substances.

To assemble biosensors, many different materials have been considered as mediators of electron transfer or analyte–receptors. Pillar[n]arenes, which consist of hydroxyquinone or its derivatives, linked by methylene bridges at 2,5-positions, are one of the prospect modifiers mentioned [7]. They have a rigid architecture and hydrophobic electron-donating cavity that can bind various analyte molecules according to the “guest-host” principle. This allows for their use in various nanocomposites [8,9], in molecular recognition [10,11], chemosensor assembling [12,13], ion transportation [14], and the assembling of supramolecular polymers [15]. Literature reports the use of pillar[n]arenes in smart materials [16], drug release systems [17], as adsorbents [18], in chemical catalysis [19] and, most widely, in chemical sensors, because pillar[5]arenes are electrochemically active and exhibit mediator properties in electrode reactions [20].

Calix[n]arenes are another class of macrocyclic compounds frequently used in sensor development. They have attracted much interest due to their ability to form host–guest complexes and act as receptors for cations, anions or neutral molecules interacting with functional groups of substituents at upper and lower rims of the macrocycles [21,22]. The formation of various stereoisomeric forms and simple functionalization make calixarenes a universal building platform for the creation of receptors, extractants, and colloidal systems sensitive to external stimuli [23,24]. Thiacalix[4]arenes are the most actively used among the derivatives of calix[4]arenes [25]. Four sulfur atoms replacing methylene linkers lead to changes in the properties of the macrocycle. Namely, the size of the cavity increases. The modification becomes easier, and the structure becomes more flexible with a wider range of conformational configurations [26]. There are numerous cases of the application of calix[n]arenes and thiacalix[4]arenes as parts of electrochemical sensors, e.g., in DNA sensors [27], those for the determination of metal ions [28], dopamine [29] and serotonin [30].

Electroactive polymers, synthesized directly on the surface of electrodes from phenothiazine dyes by electropolymerization, combine mediator properties [31] and the ability to collect analytes due to non-covalent interactions and their inclusion in the polymer matrix. Phenothiazine dyes are among the best candidates for use in electrochemical biosensors due to the variety of electrochemical activities associated with the heteroaromatic core and the electron-donating properties of the sulfur heteroatom [32]. In addition, the polymer materials promote a reduced influence of the interferences, when signals are measured directly in the sample of interest, and the expansion of the linear range of the concentrations determined with biosensors [33]. To increase the efficiency of electrochemical polymerization, a preliminary modification of monomers can be carried out [34]. The efficiency of polymeric forms of the phenothiazine dyes in the assembly of biosensors has been proved on the example of the immobilization of glucose oxidase and uricase on the poly(thionine) film [35].

The combination of the modifiers described above in the composition of electrochemical sensors can increase sensitivity and reduce LOD for the determination of analytes.

Uric acid (7,9-dihydro-1H-purine-2,6,8(3H)-trione) is the final metabolite of purines in humans and an important clinical biomarker [36]. Normal serum uric acid concentration is 149–416 µM for men and 89–357 µM for women [37]. An increase in uric acid levels above 420 µM indicates hyperuricemia [38] and can be a symptom of gout, pneumonia [39], and leukemia [40]. Therefore, the determination of uric acid in biological fluids can be used for early diagnosis of the above-mentioned diseases. It is determined by various traditional methods, i.e., high-performance liquid chromatography [41], colorimetry [42], and chemiluminescent capillary electrophoresis [43]. However, these methods require time-consuming sample preparation and are available only in specialized laboratories equipped with expensive instruments.

In this work, we have developed a flow-through biosensor system for the determination of uric acid based on a compact electrochemical cell with a replaceable reactor. Uricase was immobilized on its inner surface by carbodiimide binding. The cell was designed and manufactured by 3D printing from poly(lactic acid). This cell is an improved version of the cell from the flow-through system previously presented in [44,45]. The new design changed the flow cell toward miniaturization, easier manufacture, maintenance, replacement of structural parts, and, as a result, simpler application for the end-user. SPEs modified with carbon black (CB), pillar[5]arene, PAMAM-calix-dendrimers, and poly(arylphenothiazine) were used as a sensing element of the system. The modification of the electrode significantly increased sensitivity and decreased the LOD of uric acid.

## 2. Materials and Methods

### 2.1. Reagents

**Figure 1:**
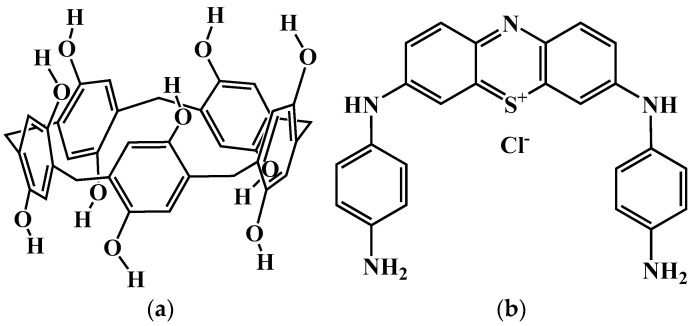
Structural formulae of: (**a**) pillar[5]arene; (**b**) PhTz-(NH_2_)_2_.

**Figure 2:**
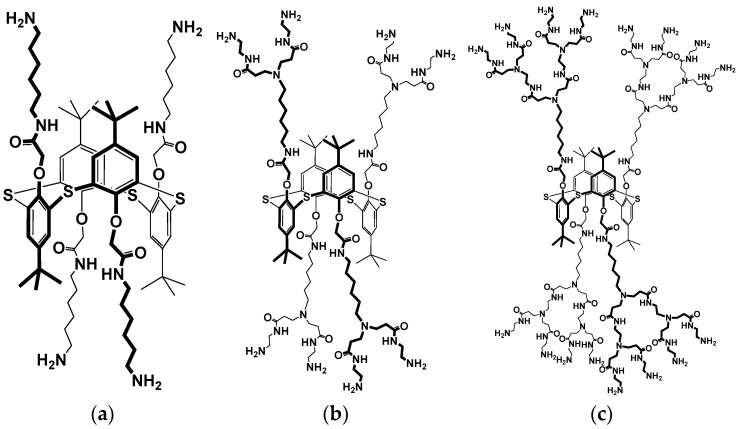
Structural formulae of PAMAM-calix-dendrimers (**a**) G0; (**b**) G1; (**c**) G2.

Uricase from *Candida* sp. (EC 1.7.3.3, lyophilized powder, ≥2 U/mg solid, Product No. U0880), polyallylamine, uric acid, poly(lactic acid), *N*-(3-dimethylaminopropyl)-*N*’-ethylcarbodiimide chloride (EDC), *N*-hydroxysuccinimide (NHS) were purchased from Sigma-Aldrich (St. Louis, MO, USA). Pillar[5]arene (Figure 1a) [46], 3,7-bis(4-aminophenylamino) phenothiazin-5-ium chloride (PhTz-(NH_2_)_2_) (Figure 1b) [47], PAMAM-calix-dendrimers G0 [48], G1 [49], G2 [50] (Figure 2) were synthesized using literature methods. CB (ENSACO 250G, >99.95% C) was purchased from Imerys Graphite & Carbon (Willebroek, Belgium).

All working solutions were prepared using Millipore Q^®^ water (Simplicity^®^ water purification system, Merck-Millipore, Molsheim, France). Other reagents were of analytical grade.

Artificial urine contained 10 mM CaCl_2_, 6 mM MgCl_2_, 6 mM Na_2_SO_4_, 2 mM potassium citrate, 20 mM KH_2_PO_4_, 21 mM KCl, 18 mM NH_4_Cl, 9 mM creatinine and 416 mM urea [51].

Electrochemical measurements were performed in 0.01 M phosphate buffer containing 0.1 M NaCl.

### 2.2. Screen-Printed Electrodes Manufacture and Modification

**Figure 3:**
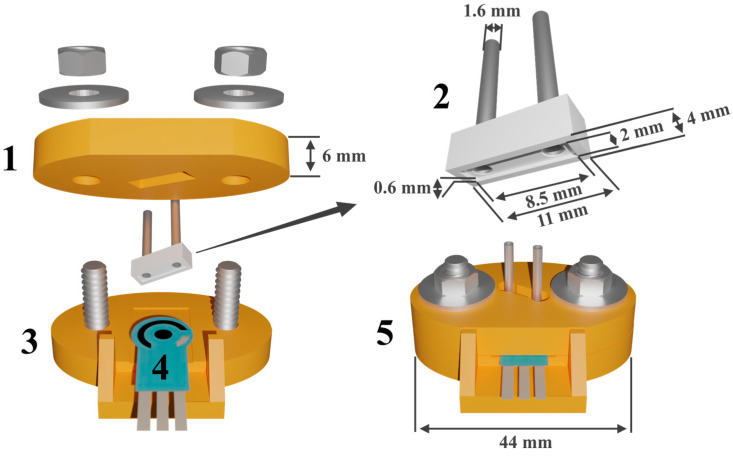
Flow-through cell design with the SPE set: (**1**) top part; (**2**) replaceable reactor with inlet and outlet stainless needles; (**3**) bottom part; (**4**) SPE set; (**5**) assembled cell with the SPE set.

SPEs were produced on the DEC 248 printer (DEK, London, UK) as described elsewhere [45]. The electrode set made on a polycarbonate sheet had dimensions of 11 × 27 mm and contained a working electrode with a working area of 3.8 mm^2^, an auxiliary electrode, and a Ag pseudo-reference electrode (see Figure 3(4)). Modification of the working electrode was carried out by dropping onto it 1 μL of a propylene carbonate suspension containing 0.66 mg·mL^−1^ CB (pre-oxidized with nitric acid) and 10 mM pillar[5]arene. It was then dried at 100 °C. On the top of the resulting layer, 1 μL of 1 mM PAMAM-calix-dendrimer in ethanol was cast and the electropolymerization of 0.1 mM PhTz-(NH_2_)_2_ was carried out. The electropolymerization was performed by multiple cycling of the potential in the conditions established previously [52]. Fifteen potential sweep cycles were applied.

### 2.3. Design and Mounting of Flow-Through Cell and Uricase Immobilization

The flow-through cell was prepared using Wanhao Duplicator 9/300 (Jinhua Wanhao Spare Parts Co., Wanhao, Hangzhou, China) with single extruder (nozzle diameter 0.3 mm) from the poly(lactic acid) filaments. Layer thickness was 0.1 mm and printing rate 70 mm s^−1^. The printing temperature was 220 °C. The design of the flow-through cell is shown in Figure 3. The cell consisted of two components secured by two screws with corresponding flat washers and nuts. At the base of the cell, there was a rectangular cutout for fixing the electrode. The rectangular reactor contained two channels equipped with plastic tubes with stainless needles for pumping solutions through the cell. The reactor was placed in a rectangular cutout in the cell lid, positioned at an angle of 30° to ensure precise placement of the reactor chamber on the working electrode. A cavity at the bottom of the reactor, 0.6 mm deep and 17 mm^2^ in area, served as a working cell. The inner surface of the cavity was used to immobilize the enzyme. This cell is an improved version of the flow-through cell with a replaceable reactor that previously showed its efficiency in biosensor designs [44,45]. To immobilize uricase, the reactor was first fixed upside down. Then, 5 μL of 100 mM EDC and 5 μL of 400 mM NHS were dropped onto the inner surface of the reactor. After a 10-min. incubation, the reactor was washed with deionized water. After that, 5 μL of the enzyme solution containing 5 U of uricase were placed into the same cavity and left at room temperature for 60 min. The immobilization occurred through the carbodiimide reaction with a formation of covalent bonds between activated terminal carboxyl groups of poly(lactic acid) of the reactor and the amino groups of enzyme molecules. Finally, the reactor was carefully washed with deionized water.

### 2.4. Biosensor Signal Measurement

The flow-through cell with SPE was assembled, then the buffer or a solution containing uric acid were alternately pumped through it using a Model 100 syringe pump (ALS, Tokyo, Japan). Currents corresponding to the reduction of the enzymatic reaction products were recorded in chronoamperometric mode using a BioStat multichannel potentiostat with dedicated software BIOSTAT version 2.1.3 (ESA Biosciences, Inc., Chelmsford, MA, USA). Cyclic voltammograms were recorded using the CHI 660E electrochemical workstation with dedicated software CHI version 14.08 (CH Instruments, Austin, TX, USA). Statistical treatment of the results was carried out using the OriginPro 8.1 application (OriginLab Corp., Northampton, MA, USA).

## 3. Results

### 3.1. Electrochemical Properties of PhTz-(NH_2_)_2_ Polymerized on Modified SPE

Phenothiazine dyes have shown their effectiveness for use in electrochemical biosensors [53,54,55]. The functionalization of these compounds with additional groups changes their properties and can affect the performance of appropriate sensors. The formation of electropolymerization products of PhTz-(NH_2_)_2_ has been previously reported [52]. The electropolymerization of phenothiazines involves depositing a dense thin film with high adhesion to the electrode surface that exhibits a significant redox response in a certain potential range.

**Figure 4:**
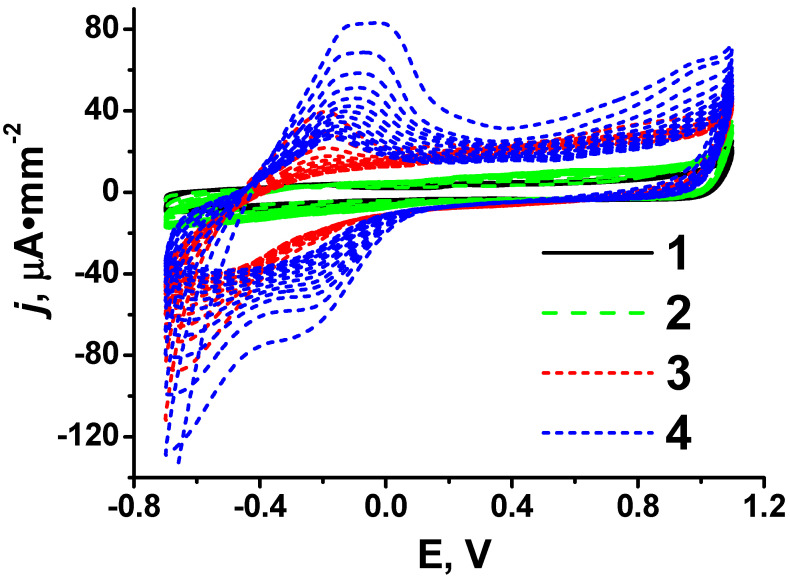
Cyclic voltammograms, 15 cycles, recorded in the presence of 0.1 mM PhTz-(NH_2_)_2_ during electropolymerization on SPE modified with: (**1**) unmodified SPE; (**2**) CB; (**3**) CB and pillar[5]arene; (**4**) CB, pillar[5]arene and PAMAM-calix-dendrimer G2. Measurements in 0.01 M phosphate buffer + 0.1 M NaCl, pH = 7.0, 100 mV/s.

To increase the efficiency of the PhTz-(NH_2_)_2_ electropolymerization, the electrodes were preliminarily modified with pillar[5]arene and PAMAM-calix-dendrimers. Pillar[5]arene has proven to be an effective electron transfer mediator. However, direct adsorption of pillar[5]arene onto a carbon electrode leads to its rapid inactivation due to the chemisorption of intermediate oxidation products [56]. Therefore, it is usually included in the surface layer together with a carbon material that acts as a carrier of the macrocycle and increases the effective surface area of an electrode. We used CB as a carbon matrix. To increase its dispersibility, CB was pre-oxidized and dispersed in propylene carbonate together with pillar[5]arene. The use of propylene carbonate made it possible to apply pillar[5]arene together with CB from a single aliquot according to the “one-pot synthesis” approach. Fifteen cycles of the potential were applied in the stage of the PhTz-(NH_2_)_2_ electropolymerization performed in the presence of pillar[5]arene. The anodic currents in the potential range from −0.4 to 0.2 V and cathodic currents in the potential range from −0.1 to −0.7 V were more than three times greater than those obtained with the coating without pillar[5]arene. This can be due to reversible oxidation of pillar[5]arene together with the products of the electropolymerization (Figure 4). To increase the efficiency of electropolymerization, PAMAM-calix-dendrimers were implemented into the composition of the surface layer. Changes in cyclic voltammograms during the electropolymerization of PhTz-(NH_2_)_2_ resulted from the additional modification of the electrodes with PAMAM-calix-dendrimer G2 (Figure 2c), as shown in Figure 4(4).

**Figure 5:**
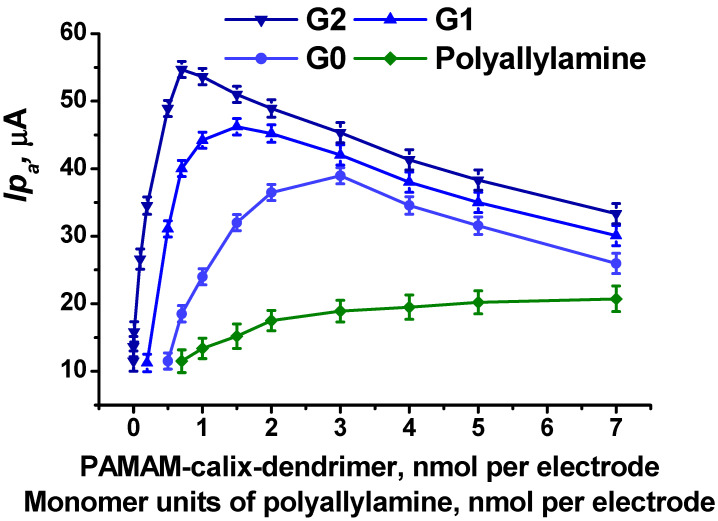
The dependences of anodic peak currents for PhTz-(NH_2_)_2_, electropolymerized onto SPE modified with CB, pillar[5]arene, a PAMAM-calix-dendrimer or polyallylamine, on the amount of PAMAM-calix-dendrimer and polyallylamine (expressed in moles of monomer) used for modification. Anodic peak currents measured in the potential range from −0.4 to 0.2 V. Average ± S.D. of five individual electrodes. Measurements in 0.01 M phosphate buffer + 0.1 M NaCl, pH = 7.0, 100 mV·s^−1^.

The implementation of the PAMAM-calix-dendrimers into the surface layer increased the peak currents of the electropolymerized PhTz-(NH_2_)_2_. Moreover, the peak currents grew with the increasing generation of the number of the PAMAM-calix-dendrimers from G0 to G2 (Figure 5). In comparison, similar experiments were also performed with polyallylamine containing the same functional primary amino groups. Figure 5 shows the dependence of peak currents on the amount of PAMAM-calix-dendrimers and polyallylamine used for the modification of the surface layer. For each of the PAMAM-calix-dendrimers, a maximum was observed on the dependence. With further increase in the amount of the PAMAM-calix-dendrimers per electrode, the peak currents began to decrease. This can be due to the physical blocking of the working surface of the electrode with non-conducting PAMAM-calix-dendrimer molecules. In the case of the PAMAM-calix-dendrimer G2, the currents of the polymer coating increased more than fivefold compared to the coating with no PAMAM-calix-dendrimer. Figure 5 shows that the current increased from 11 to 54 µA when using the electrode modification with 1 nmol of the PAMAM-calix-dendrimer G2. It is worth noting that the introduction of polyallylamine as a polycation instead of PAMAM-calix-dendrimers did not result in noticeable changes in the peak currents. This fact indicates that the influence of PAMAM-calix-dendrimers can be explained by the structure of the PAMAM-calix-dendrimer molecules but not by the charge of amino groups.

**Figure 6:**
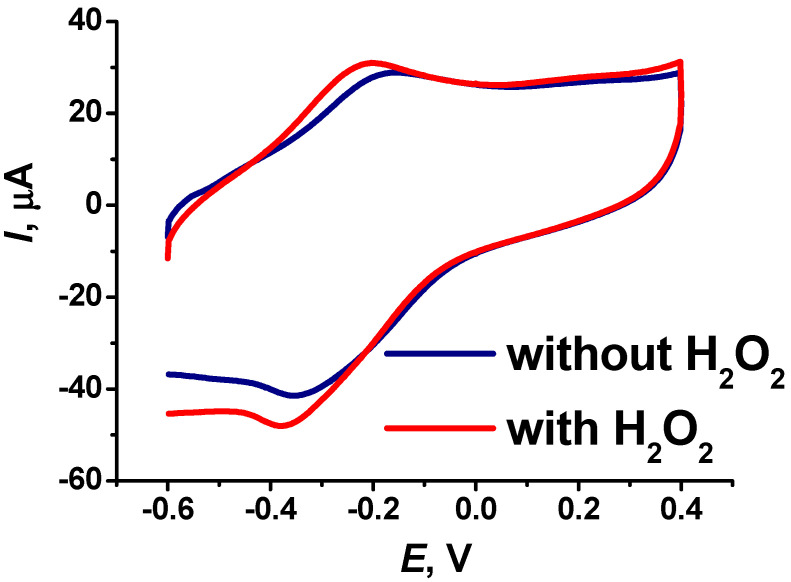
Cyclic voltammograms obtained in the absence and in the presence of 0.1 mM hydrogen peroxide on the SPE modified with CB, pillar[5]arene, PAMAM-calix-dendrimer G2 and electropolymerized PhTz-(NH_2_)_2_. Measurements in 0.01 M phosphate buffer + 0.1 M NaCl, pH = 7.0, 100 mV·s^−1^.

The resulting electrode coating demonstrated sensitivity to hydrogen peroxide, which was used for further development of the biosensor system. Figure 6 shows cyclic voltammograms obtained in a stationary solution (with stopped flow) using the modified electrode in the presence, and in the absence, of hydrogen peroxide. The presence of hydrogen peroxide increased the cathodic peak near −0.4 V.

### 3.2. Flow-Through Uricase Biosensor System

To assemble the flow-through biosensor system, SPE modified with CB, pillar[5]arene, PAMAM-calix-dendrimer and electropolymerized PhTz-(NH_2_)_2_ was used. Uricase was immobilized to the inner side of the replaceable reactor (Figure 3(2)) by carbodiimide binding. The design of the biosensor system ensures the separation of the immobilization site from the electrode modification site. This makes the system more flexible for the enzyme replacement and further modifications of the electrode as a primary transducer of a biosensor.

In the presence of uric acid, hydrogen peroxide is formed on the inner walls of the reactor (Equation (1)). Then, it is cathodically reduced on the electrode located at the bottom of the reactor chamber inside the flow cell.

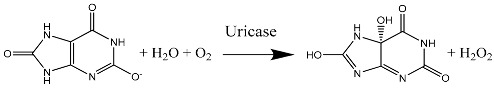
(1)


**Figure 7:**
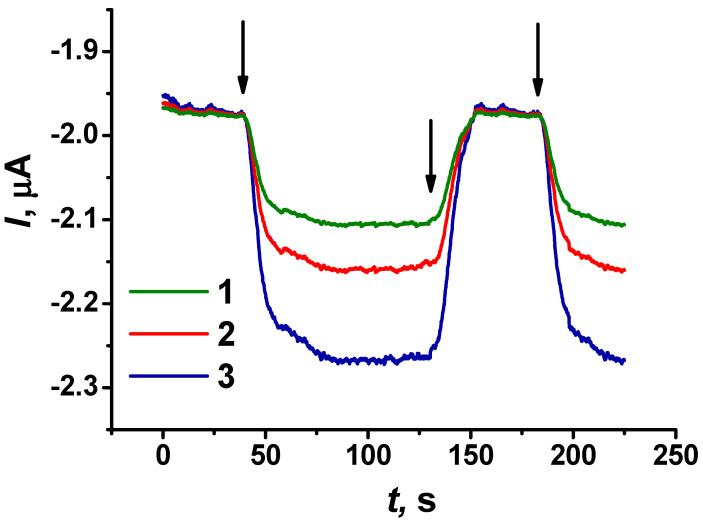
Chronoamperometric response recorded with the flow-through biosensor system to 20 µM uric acid using differently modified SPEs: (1) CB + pillar[5]arene, −0.45 V; (2) CB + pillar[5]arene + poly(PhTz-(NH_2_)_2_), −0.35 V; (3) CB + pillar[5]arene + PAMAM-calix-dendrimer G2 + poly(PhTz-(NH_2_)_2_), −0.35 V. Measurements in 0.01 M phosphate buffer + 0.1 M NaCl, pH = 8.0. Arrows indicate switching the flow of the buffer solution to the uric acid solution and vice versa.

The electrochemical response of the sensor to uric acid was measured in chronoamperometric mode by switching the flow of the phosphate buffer and that of uric acid. The uric acid signal was measured at a constant potential as the maximum change in the current after the flow switching. Examples of the chronoamperometric response of the biosensor system to uric acid are presented in Figure 7 for differently modified electrodes.

**Figure 8:**
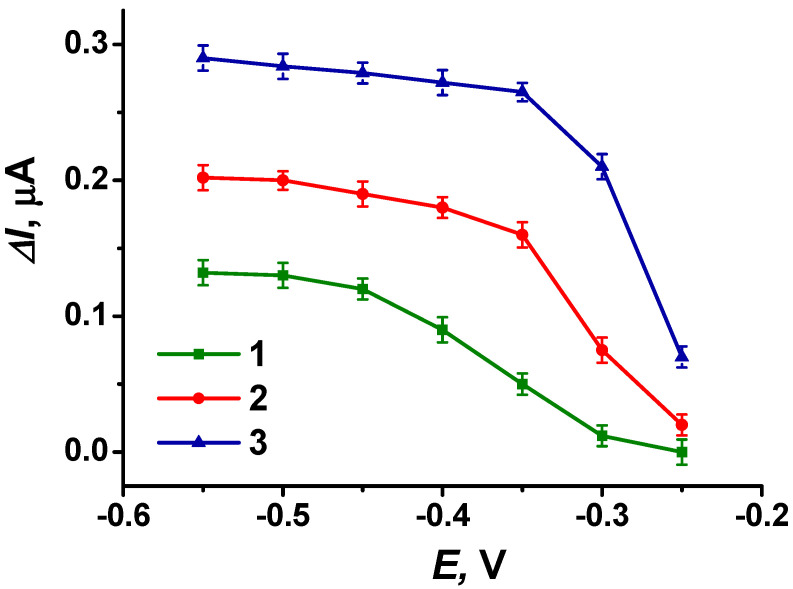
The dependences of response of the flow-through biosensor system to 20 µM uric acid on the potential applied to differently modified SPEs: (1) CB + pillar[5]arene; (2) CB + pillar[5]arene + poly(PhTz-(NH_2_)_2_; (3) CB + pillar[5]arene + PAMAM-calix-dendrimer G2 + poly(PhTz-(NH_2_)_2_. Measurements in 0.01 M phosphate buffer + 0.1 M NaCl, pH = 8.0.

Figure 8 shows the dependence of the recorded response of the biosensor system to uric acid on the electrode potential obtained from the electrodes with different modifications. The maximum response was observed for the electrode modified with CB, pillar[5]arene, PAMAM-calix-dendrimer G2 and poly(PhTz-(NH_2_)_2_). For it, a signal close to the maximum can be measured at −0.35 V, which was selected for use in further experiments.

Optimal operation conditions corresponding to the maximal response were established for the biosensor system. The minimal amount of uricase used for immobilization to produce a stable and reproduceable response to the uric acid was 5 U per reactor (Figure S1). The maximum response was observed at phosphate buffer pH = 8.0 (Figure S2). The signal also increased with the flow rate until stabilization at about 0.2 mL·min^−1^ (Figure S3).

**Figure 9:**
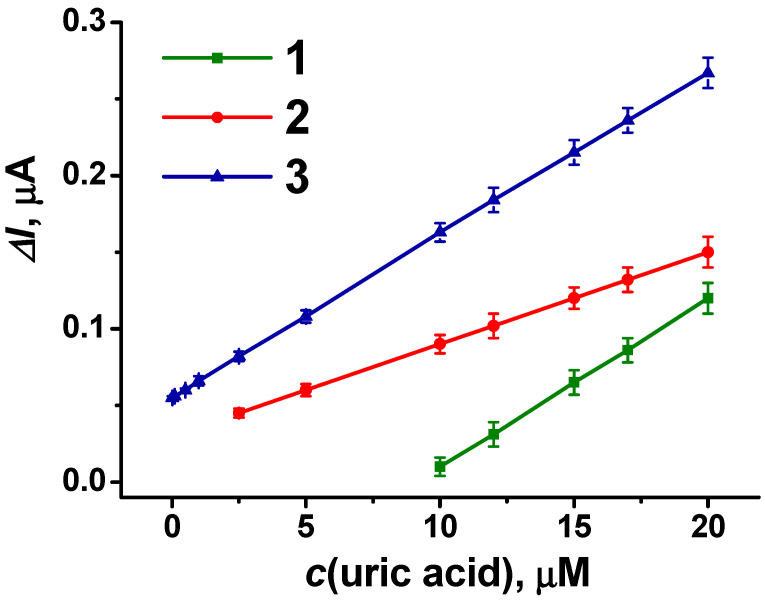
The dependences of the response of the flow-through biosensor system on the uric acid concentration obtained with differently modified SPEs: (1) CB + pillar[5]arene, −0.45 V; (2) CB + pillar[5]arene + poly(PhTz-(NH_2_)_2_), −0.35 V; (3) CB + pillar[5]arene + PAMAM-calix-dendrimer G2 + poly(PhTz-(NH_2_)_2_), −0.35 V. Measurements in 0.01 M phosphate buffer + 0.1 M NaCl, pH = 8.0.

The dependences of the biosensor system signal on the concentration of uric acid in the buffer solution obtained under optimal measurement conditions are presented in Figure 9 for variously modified electrodes. It can be seen that the presence of PAMAM-calix-dendrimer G2 in the coating increased the redox signal related to the uric acid assay by more than 1.5 times (dependence 3 compared to 2).

The highest sensitivity and minimal LOD were achieved with the SPEs modified with CB, pillar[5]arene, PAMAM-calix-dendrimer G2 and poly(PhTz-(NH_2_)_2_). Parameters of the linear regression equation are as follow: Δ*I*, μA = (0.055 ± 0.001) + (10.69 ± 0.03) × (*c*, mM), *R*^2^ = 0.9998. This biosensor system allows for the determination of uric acid in the concentration range from 10 nM to 20 µM with the LOD of 4 nM, calculated by 3 σ/*S* (where σ is the standard deviation of background measurements (n = 5) and *S* is the slope of the calibration curve).

The analytical performance of the developed biosensor system for uric acid determination is better than or comparable with those previously reported for electrochemical sensors (Table 1). It should be noted that the use of such sensors in medicine requires measuring much higher levels of uric acid. Taking this into account, lower concentrations in the determination range allow for a significant dilution of the analyzed biological sample to level out the interfering influence of matrix components. The time for one measurement of a uric acid concentration is about 3 min.

The accuracy of the uric acid determination was assessed in a series of five consecutive measurements with the same reactor and electrode. The standard deviation of the signal for 10 µM uric acid for the same reactor was 2.1%, while for five replacement reactors it increased to 5.5%. The immobilized enzyme maintained its activity in a continuous flow for at least 8 h (Figure S4). As it had been shown earlier for the same reactor and immobilization procedure of uricase [44], 50% of the enzyme activity was retained six months after immobilization when stored in dry conditions.

One of the advantages of measuring the cathode current was the absence of the influence of oxidizable substances on the biosensor signal. The following potential interfering substances were tested (maximum concentrations without effect are presented): glucose 1 mM, dopamine 0.5 mM, and ascorbic acid 50 μM (Figure S5).

The system was tested on artificial urine samples (Table 2). The degree of discovery was 122% for an undiluted solution and 101% for a tenfold dilution of the original sample with the phosphate buffer solution. Considering the normal content of uric acid in biological fluids, the developed biosensor system provides a reliable determination of the biomarker, sufficient for the early diagnosis of relevant diseases.

## 4. Discussion

A flow-through biosensor system with uricase immobilized on the inner surface of the 3D-printed replaceable poly(lactic acid) reactor was developed. The design of the flow-through cell was significantly improved over the previously presented cell [44,45] by reduced working volume, shorter tubing, and better compliance of the geometry with the SPE applied as signal transducer. Physical separation of the biocomponent immobilized on the surface of the poly(lactic acid) reactor from the transducer, a modified SPE, allows for the minimization of the aggressive effects of the reagents used for the enzyme immobilization and electrode modification. The SPE used were modified with CB, pillar[5]arene, PAMAM-calix-dendrimer G2 and poly(PhTz-(NH_2_)_2_). The modification increased the sensitivity of the electrode signal recorded in chronoamperometric mode toward hydrogen peroxide, a product of the enzymatic oxidation of uric acid. The performance of the developed biosensor system was comparable or better than previously reported for similar electrochemical biosensors. The use of cathode current as analytical signal made it possible to avoid the interfering influence of most substances prone to oxidation. These factors make the developed flow-through biosensor system promising for the inexpensive but reliable monitoring of metabolites in biological fluids. If necessary, the system can be easily adapted to other enzymes or modified for a point-of-care testing format. The low flow rate, small reactor volume, and low-cost manufacture make the biosensor system quite competitive with more complex microfluidic devices or implantable biosensors.

## Figures and Tables

**Table 1:** Uric acid determination with electrochemical sensors.

Electrode and Modifications	Method of Measurement	Concentration Range, M	LOD, M	Reference
Glassy carbon electrode, nitrogen-doped graphene aerogel	Cyclic voltammetry	4 × 10^−7^–5 × 10^−5^	1.2 × 10^−7^	[57]
Gold electrode, carbon nanotubes, carboxymethylcellulose	Cyclic voltammetry	2 × 10^−5^−2.7 × 10^−3^	2.8 × 10^−6^	[58]
Zeolite imidazolate framework-11 modified electrode	Differential pulse voltammetry	5 × 10^−6^−5.4 × 10^−4^	4.8 × 10^−7^	[59]
Ultrasmall iron oxide nanoparticles decorated urchin-like nitrogen-doped carbon	Differential pulse voltammetry	2 × 10^−6^–2 × 10^−4^	2.9 × 10^−7^	[60]
Covalent organic frameworks and Ox-MWCNT Co-Modified glassy carbon electrode	Differential pulse voltammetry	6 × 10^−7^−2.5 × 10^−4^	6.3 × 10^−8^	[61]
SPE, pillar[5]arene, poly(methylene blue), polythionine, uricase on polylactic acid	Chronoamperometry in flow-through conditions	1 × 10^−7^–1 × 10^−5^	3 × 10^−8^	[44]
SPE, CB, pillar[5]arene, PAMAM-calix-dendrimer G2, poly(PhTz-(NH_2_)_2_),uricase on polylactic acid	Chronoamperometry in flow-through conditions	1 × 10^−8^–2 × 10^−5^	4 × 10^−9^	This work

**Table 2:** Uric acid determination and recovery measured in spiked samples of artificial urine.

Measurement Medium	Spiked, μM	Found, μM	Recovery, %
Undiluted artificial urine	10	12.2 ± 0.2	122 ± 2
3 times diluted artificial urine	10	10.7 ± 0.1	107 ± 1
10 times diluted artificial urine	10	10.1 ± 0.1	101 ± 1

## Data Availability

Data is contained within the article or Supplementary Material.

## Supplementary Materials

The following supporting information can be downloaded at: https://www.mdpi.com/article/10.3390/bios14030120/s1, Figure S1: The dependence of response of the flow-through biosensor system to 20 µM uric acid on amount of uricase used for immobilization. The data obtained with SPE modified with CB + pillar[5]arene + PAMAM-calix-dendrimer G2 + poly(PhTz-(NH_2_)_2_), potential −0.35 V, flow rate 0.2 mL·min^−1^. Measurements in 0.01 phosphate buffer + 0.1 M NaCl, pH = 8.0; Figure S2: The dependence of response of the flow-through biosensor system to 20 µM uric acid on pH of solution. The data obtained with SPE modified with CB + pillar[5]arene + PAMAM-calix-dendrimer G2 + poly(PhTz-(NH_2_)_2_), potential −0.35 V, flow rate 0.2 mL·min^−1^. Measurements in 0.01 phosphate buffer + 0.1 M NaCl; Figure S3: The dependence of response of the flow-through biosensor system to 20 µM uric acid on flow rate of solution. The data obtained with SPE modified with CB + pillar[5]arene + PAMAM-calix-dendrimer G2 + poly(PhTz-(NH_2_)_2_), potential −0.35 V. Measurements in 0.01 phosphate buffer + 0.1 M NaCl, pH = 8.0; Figure S4: Time dependency of response of the flow-through biosensor system to 10 µM uric acid. The data obtained with SPE modified with CB + pillar[5]arene + PAMAM-calix-dendrimer G2 + poly(PhTz-(NH_2_)_2_), potential −0.35 V, flow rate 0.2 mL·min^−1^. Measurements in 0.01 phosphate buffer + 0.1 M NaCl, pH = 8.0. The mean and standard deviation values for 5 electrodes are presented; Figure S5: Influence of interfering substances to response of the flow-through biosensor system measured in presence of 10 µM uric acid and 1—No interfering substance, 2—Glucose 1 mM, 3—Ascorbic acid 50 μM, 4—Dopamine 0.5 mM. The data obtained with SPE modified with CB + pillar[5]arene + PAMAM-calix-dendrimer G2 + poly(PhTz-(NH_2_)_2_), potential −0.35 V, flow rate 0.2 mL·min^−1^. Measurements in 0.01 phosphate buffer + 0.1 M NaCl, pH = 8.0. The mean and standard deviation values for 5 electrodes are presented.
