# Supplementary material for: Flow-Through Amperometric Biosensor System Based on Functionalized Aryl Derivative of Phenothiazine and PAMAM-Calix-Dendrimers for the Determination of Uric Acid
Source: Biosensors (Basel). 2024 Feb 23;14(3):120. doi: 10.3390/bios14030120 (PMC10968175; doi:10.3390/bios14030120)
Supplement: Supplementary file 1 [file biosensors-14-00120-s001.zip › biosensors-2828373-supplementary.pdf]

Supplementary Materials

to the article of Dmitry Stoikov, Alexey Ivanov, Insiya Shafigullina, Milena Gavrikova, Pavel Padnya, Igor Shiabiev, Ivan Stoikov and Gennady Evtugyn "Flow-through amperometric biosensor system based on functionalized aryl derivative of phenothiazine and PA-MAM-calix-dendrimers for the determination of uric acid"

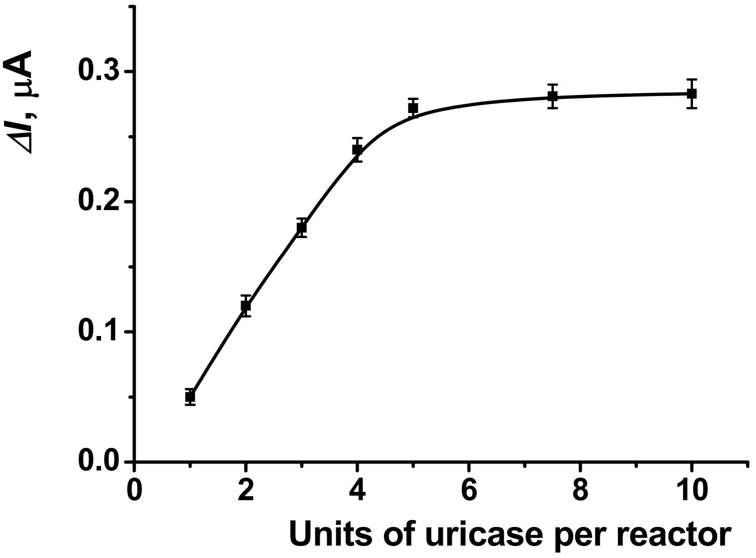

Figure S1. The dependence of response of the flow-through biosensor system to 20 μM uric acid on amount of uricase used for immobilization. The data obtained with SPE modified with CB + pillar[5]arene + PAMAM-calix-dendrimer G2 + poly(PhTz-$(NH_2)_2$), potential -0.35 V, flow rate 0.2 mL·min^{-1}. Measurements in 0.01 phosphate buffer + 0.1 M NaCl, pH = 8.0.

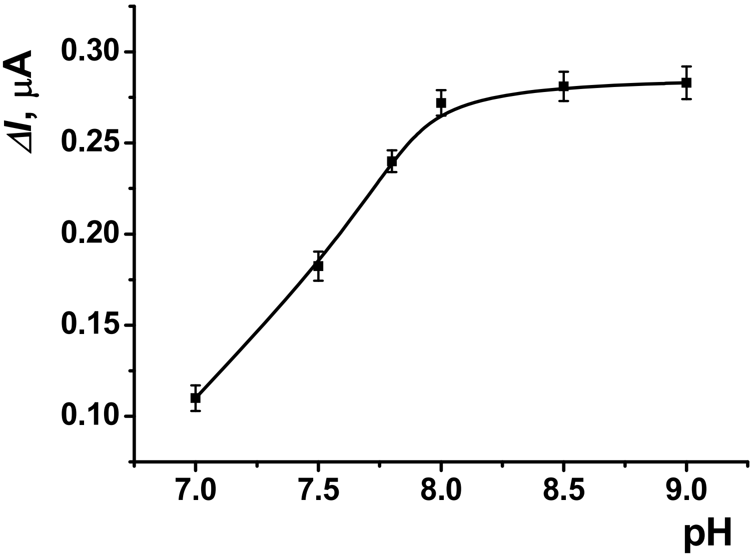

Figure S2. The dependence of response of the flow-through biosensor system to 20 μM uric acid on pH of solution. The data obtained with SPE modified with CB + pillar[5]arene + PAMAM-calix-dendrimer G2 + poly(PhTz-$(NH_2)_2$), potential -0.35 V, flow rate 0.2 mL·min^{-1}. Measurements in 0.01 phosphate buffer + 0.1 M NaCl.

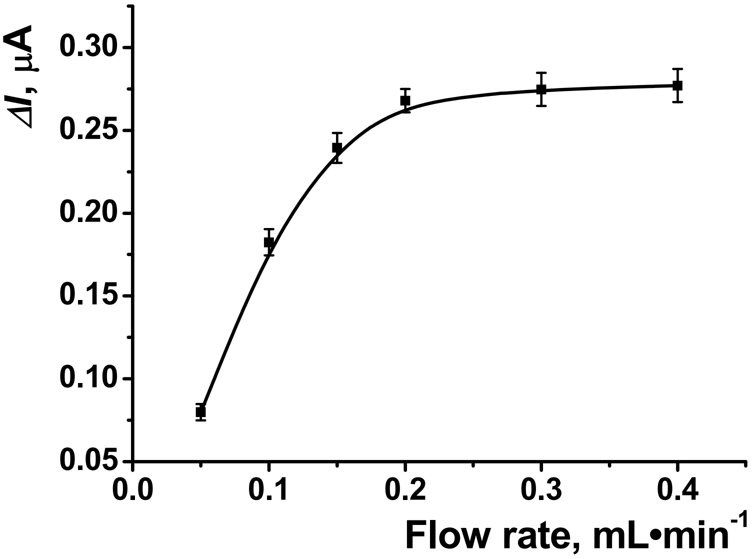

Figure S3. The dependence of response of the flow-through biosensor system to 20 μM uric acid on flow rate of solution. The data obtained with SPE modified with CB + pillar[5]arene + PAMAM-calix-dendrimer G2 + poly(PhTz-$(NH_2)_2$), potential -0.35 V. Measurements in 0.01 phosphate buffer + 0.1 M NaCl, pH = 8.0.

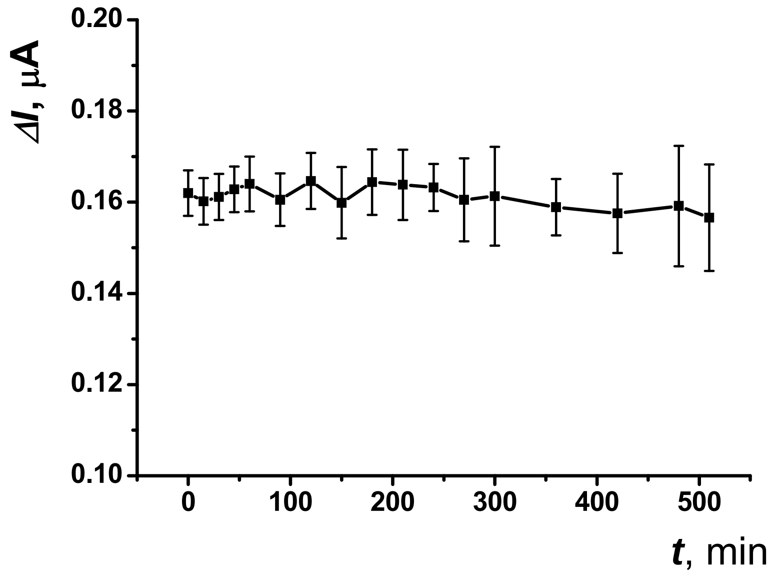

Figure S4. Time dependency of response of the flow-through biosensor system to 10 μM uric acid. The data obtained with SPE modified with CB + pillar[5]arene + PAMAM-calix-dendrimer G2 + poly(PhTz-$(NH_2)_2$), potential -0.35 V, flow rate 0.2 mL·min^{-1}. Measurements in 0.01 phosphate buffer + 0.1 M NaCl, pH = 8.0. The mean and standard deviation values for 5 electrodes are presented.

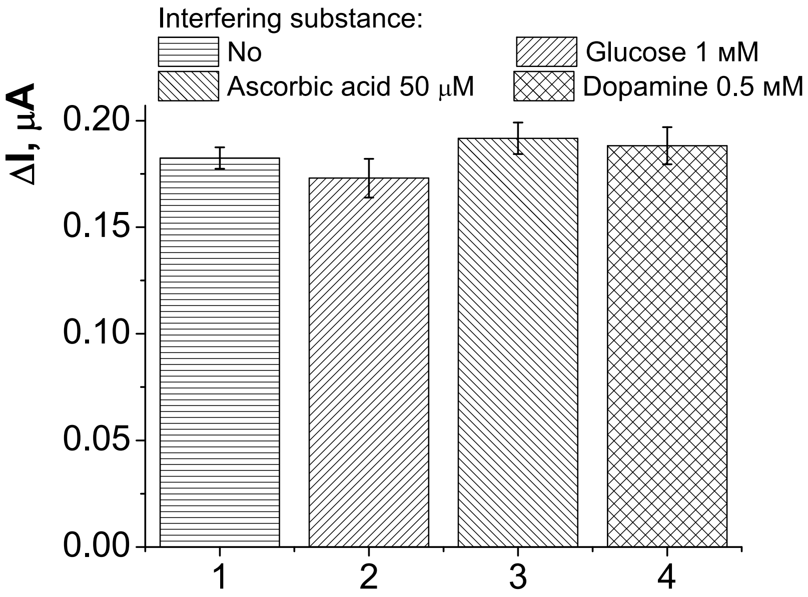

Figure S5. Influence of interfering substances to response of the flow-through biosensor system measured in presence of 10 μM uric acid and 1 – No interfering substance, 2 – Glucose 1 mM, 3 – Ascorbic acid 50 μM, 4 – Dopamine 0.5 mM. The data obtained with SPE modified with CB + pillar[5]arene + PAMAM-calix-dendrimer G2 + poly(PhTz-$(NH_2)_2$), potential -0.35 V, flow rate 0.2 mL·min^{-1}. Measurements in 0.01 phosphate buffer + 0.1 M NaCl, pH = 8.0. The mean and standard deviation values for 5 electrodes are presented.
